# Tick-Borne Encephalitis Vaccine: Recommendations of the Advisory Committee on Immunization Practices, United States, 2023

**DOI:** 10.15585/mmwr.rr7205a1

**Published:** 2023-11-10

**Authors:** Susan L. Hills, Katherine A. Poehling, Wilbur H. Chen, J. Erin Staples

**Affiliations:** ^1^Division of Vector-Borne Diseases, National Center for Emerging and Zoonotic Infectious Diseases, CDC, Fort Collins, Colorado; ^2^Wake Forest School of Medicine, Winston-Salem, North Carolina; ^3^University of Maryland School of Medicine, Baltimore, Maryland

## Abstract

**Tick-borne encephalitis (TBE) virus is focally endemic in parts of Europe and Asia. The virus is primarily transmitted to humans by the bites of infected:**

Ixodes *species ticks but can also be acquired less frequently by alimentary transmission. Other rare modes of transmission include through breastfeeding, blood transfusion, solid organ transplantation, and slaughtering of viremic animals. TBE virus can cause acute neurologic disease, which usually results in hospitalization, often permanent neurologic or cognitive sequelae, and sometimes death. TBE virus infection is a risk for certain travelers and for laboratory workers who work with the virus. In August 2021, the Food and Drug Administration approved Ticovac TBE vaccine for use among persons aged ≥1 year. This report summarizes the epidemiology of and risks for infection with TBE virus, provides information on the immunogenicity and safety of TBE vaccine, and summarizes the recommendations of the Advisory Committee on Immunization Practices (ACIP) for use of TBE vaccine among U.S. travelers and laboratory workers.*

**The risk for TBE for most U.S. travelers to areas where the disease is endemic is very low. The risk for exposure to infected ticks is highest for persons who are in areas where TBE is endemic during the main TBE virus transmission season of April–November and who are planning to engage in recreational activities in woodland habitats or who might be occupationally exposed. All persons who travel to areas where TBE is endemic should be advised to take precautions to avoid tick bites and to avoid the consumption of unpasteurized dairy products because alimentary transmission of TBE virus can occur. TBE vaccine can further reduce infection risk and might be indicated for certain persons who are at higher risk for TBE. The key factors in the risk-benefit assessment for vaccination are likelihood of exposure to ticks based on activities and itinerary (e.g., location, rurality, season, and duration of travel or residence). Other risk-benefit considerations should include 1) the rare occurrence of TBE but its potentially high morbidity and mortality, 2) the higher risk for severe disease among certain persons (e.g., older persons aged ≥60 years), 3) the availability of an effective vaccine, 4) the possibility but low probability of serious adverse events after vaccination, 5) the likelihood of future travel to areas where TBE is endemic, and 6) personal perception and tolerance of risk:**

**ACIP recommends TBE vaccine for U.S. persons who are moving or traveling to an area where the disease is endemic and will have extensive exposure to ticks based on their planned outdoor activities and itinerary. Extensive exposure can be considered based on the duration of travel and frequency of exposure and might include shorter-term (e.g., <1 month) travelers with daily or frequent exposure or longer-term travelers with regular (e.g., a few times a month) exposure to environments that might harbor infected ticks. In addition, TBE vaccine may be considered for persons who might engage in outdoor activities in areas where ticks are likely to be found, with a decision to vaccinate made on the basis of an assessment of their planned activities and itinerary, risk factors for a poor medical outcome, and personal perception and tolerance of risk. In the laboratory setting, ACIP recommends TBE vaccine for laboratory workers with a potential for exposure to TBE virus:**

## Introduction

Tick-borne encephalitis (TBE) virus is a tickborne flavivirus that is focally endemic in a geographic region extending from western and northern Europe through to northern and eastern Asia (*1*). The most recognized clinical manifestation of TBE virus infection is acute neurologic disease, which usually results in hospitalization, often permanent neurologic or cognitive sequelae, and sometimes death. TBE is rare among travelers and laboratory workers.

In August 2021, the Food and Drug Administration (FDA) approved an inactivated TBE vaccine (Ticovac) for use in persons aged ≥1 year. The current adult formulation of TBE vaccine has been used since 2001 in Europe; however, before 2021, no TBE vaccine was licensed in the United States, and the Advisory Committee on Immunization Practices (ACIP) had no recommendations for use of TBE vaccine (*2*,*3*). This report provides information on TBE and TBE vaccine and describes the new ACIP recommendations for use of TBE vaccine among U.S. travelers and laboratory workers. Health care providers can use these guidelines to discuss the risks and benefits of TBE vaccination during pretravel consultations with persons traveling abroad to areas with TBE virus transmission risk and in occupational health consultations with laboratory workers with potential for exposure to TBE virus. The TBE vaccine recommendations will be reviewed and updated as needed if new data become available or if additional TBE vaccines are licensed in the United States.

## Background

### TBE Virus and Its Subtypes

TBE virus is a single-stranded RNA virus in the genus *Flavivirus,* family Flaviviridae (*4*). TBE virus is closely related to Powassan virus, a tickborne flavivirus transmitted in parts of the United States (*5*). The three main antigenic subtypes of TBE virus (i.e., European, Siberian, and Far Eastern) differ in the severity of disease they cause and geographic distribution (*6*). The principal geographic distribution of the European subtype virus is in parts of western and northern Europe through to the eastern European countries; the Siberian subtype virus is in Siberia and the Ural and European parts of Russia; and the Far Eastern subtype virus is in Japan, China, Mongolia, and the eastern parts of Russia; however, subtype virus distributions overlap substantially (*6*–*9*). Genomic studies have indicated two additional minor subtype viruses (i.e., Baikalian and Himalayan) (*10*,*11*).

### Modes of TBE Virus Transmission

TBE virus is primarily transmitted to humans by the bites of infected *Ixodes* sp. ticks but can also be acquired less frequently by alimentary transmission. Other rare modes of transmission include through breastfeeding, blood transfusion, solid organ transplantation, and slaughtering of viremic animals. Nymphs and adult ticks are believed to be responsible for causing most human infections. Approximately 60%–70% of persons with TBE recall a bite (*12*–*19*). Because TBE virus is present in the saliva of an infected tick, transmission likely occurs early during feeding (*1*,*20*).

#### Transmission by Ticks

*Ixodes ricinus* is the main vector for the European subtype TBE virus and *Ixodes persulcatus* for the Siberian and Far Eastern subtype viruses (*21*). *I. ricinus* is found in most of continental Europe and the United Kingdom and *I. persulcatus* in an area extending east from northeastern Europe through to China and Japan (*22*,*23*). The distributions of the two species overlap in certain countries, including Estonia, Finland, Latvia, and the European part of Russia (*7*,*21*,*23*–*27*).

The preferred habitats for the vector ticks are woodland environments. The main habitats are deciduous forests for *I. ricinus* and coniferous forests for *I. persulcatus* (*23*,*28*). Ticks can be found either within the forest or on forest edges, where the forest transitions to grasslands, meadows, or marshlands, and they favor areas with low-growing dense brush and plant litter (*23*,*29*–*32*). Recreational activities with increased risk for exposure to ticks include hiking, camping, cycling in woodland areas, hunting, fishing, birdwatching, and collecting mushrooms or berries (*33*–*36*). Persons in certain occupations (e.g., farmers, forestry workers, military personnel, and researchers undertaking field work in rural areas) also might be at higher risk for exposure to infected ticks (*17*,*37*,*38*). Humans must enter a tick habitat to be at risk for infection because ticks do not, unaided, disperse widely (*39*,*40*). TBE virus infections acquired in urban areas (e.g., city parks) are occasionally reported; however, risk in urban areas is considered to be low (*38*,*41*,*42*).

The enzootic transmission cycle of TBE virus involves ticks and vertebrate hosts. Ticks are both virus vectors and reservoirs. A tick can become infected when feeding on a viremic host or through nonviremic transmission when co-feeding in close proximity to an infected tick (*43*–*45*). After becoming infected, ticks remain infected through their various life stages and can transmit the virus sexually to other ticks and transovarially to their offspring (*28*,*46*,*47*). The main amplifying reservoir hosts are small mammals, particularly rodents (e.g., mice and voles). Larger forest animals (e.g., boar and deer) and domestic animals (e.g., cattle, dogs, goats, and sheep) do not have an important role in the maintenance of the virus in nature. However, deer and cattle have an important role in maintaining tick populations (*22*,*23*,*40*,*48*). Humans are incidental, dead-end hosts in the transmission cycle because they do not develop a level or duration of viremia sufficient to infect ticks or have sufficient numbers of attached ticks at one time to allow co-feeding (*22*,*49*–*51*).

#### Other Modes of Transmission

Alimentary transmission is a less frequent means of acquisition of TBE virus and occurs after ingestion of unpasteurized dairy products (e.g., milk and cheese) from infected cattle, goats, or sheep; transmission from goats is most commonly reported (*52*–*60*). Large outbreaks linked to infected dairy products have been reported from areas where TBE is endemic, including one with approximately 600 cases (*58*,*61*,*62*). Approximately 47 laboratory-acquired TBE virus infections have occurred globally (*63*–*65*). TBE virus transmission from infected breastfeeding women to their infants has been described in at least two published reports; one infant remained healthy and the other had severe sequelae (*30*,*66*). Other rare modes of transmission include blood transfusion, solid organ transplantation, and slaughtering of viremic animals (*67*–*69*).

### Epidemiology of TBE

#### Geographic Distribution and Spread

TBE virus is focally endemic in a geographic region extending from western and northern Europe through to northern and eastern Asia (https://www.cdc.gov/tick-borne-encephalitis/geographic-distribution/index.html). Although the geographic range of TBE virus is restricted by the presence of the tick vectors, areas of TBE virus transmission are more limited and focal than the tick distribution. TBE virus-infected ticks typically are found in discrete areas (i.e., foci) confined by the presence of environmental conditions that allow maintenance of the natural transmission cycle rather than being distributed evenly across a region (*13*,*70*,*71*). Natural foci can be small, with locations <1 square mile (*72*). Multiple factors are required for maintenance of virus circulation (e.g., favorable microclimatic conditions, interactions of ticks and vertebrate hosts, and local vegetation), which likely contribute to the focal occurrence (*73*). Within affected areas, tick population density and TBE virus infection rates can be highly variable. Infection rates in ticks typically range from 0.1% to 5%, although rates of approximately 40% in *I. persulcatus* ticks have been reported (*21*,*74*–*77*).

During recent decades, TBE virus has emerged in new geographical foci in countries where the disease is endemic, and the overall area of recognized transmission has expanded westward and northward (*1*,*26*,*78*–*89*). Since 2016, three countries have reported their first autochthonous cases (Belgium, England, and the Netherlands) (*90*–*92*). New TBE foci also have been detected at higher altitudes, reaching elevations up to 2,100 meters (6,890 feet) above sea level (*52*,*81*,*93*–*95*). Concurrently, in certain countries, a reduction in virus transmission and possible loss of recognized geographical foci have been documented (*81*,*93*). Various factors might be contributing to the changing distribution, including changes in climatic and ecologic conditions altering tick habitats and transmission cycles and dispersal of ticks into new areas by birds, deer, or other animals (*22*,*40*,*79*,*96*–*103*).

#### Incidence

In areas where TBE is endemic, approximately 5,000*–*10,000 new cases are reported annually (*9*,*104*). However, this figure likely represents an underestimate of the actual number of cases because of underdiagnosis, underreporting, or both in certain countries (*13*,*105*–*108*).

Incidence rates differ from country to country and depend on the local ecology and geographic distribution of the virus within the country. However, national incidence rates are not directly comparable because of variable approaches to surveillance, the extent of human and laboratory resources applied to surveillance, and the population vaccination coverage (*109*). Higher incidence rates are most commonly reported from the Baltic states (Estonia, Latvia, and Lithuania), Slovenia, and the Czech Republic (*110*).

Annual variability in countries’ incidence rates is typical (*86*,*109*,*110*). The reasons for this variability are not completely understood but reflect the complex interactions among factors that affect risk for infection, including tick density, presence of animal hosts, ecologic conditions, weather, and human behavior (*35*,*76*,*109*–*112*). Longer-term fluctuations in TBE incidence also occur (*81*). Incidence of reported cases has increased in multiple countries in recent decades while remaining stable or decreasing in others (*27*,*111*–*118*). In addition to the factors affecting annual variability in the short term, socioeconomic factors (e.g., political instability and poverty) can affect disease incidence over the longer term (*79*,*93*,*119*–*123*). Other factors that can lead to observed increases include improved awareness of TBE, increased access to laboratory diagnostics, and better surveillance (*1*,*13*,*89*,*109*,*110*,*124*). Of note, TBE became a reportable disease in the European Union in 2012 (*125*). Reduced incidence in certain countries is related to increased vaccine uptake over time; for example, in Austria, a vaccination program resulted in TBE incidence decreasing approximately sixfold from 5.7 cases per 100,000 population during 1972–1981 to 0.9 during 2002–2011 (*126*).

#### Demographic and Seasonal Patterns

TBE can occur in persons of all ages, with encephalitis reported in one infant as young as 17 days (*127*). Incidence is typically low in children and increases with age, generally peaking in the 60–69 years age group and then decreasing in the ≥70 years age group (*17*,*86*,*110*,*111*,*115*,*128*–*130*). TBE is more common in males, with reported incidence rates often 1.5–2 times higher than in females, likely reflecting a greater risk for tick exposure (*14*,*35*,*37*,*67*,*110*–*112*,*115*,*131*,*132*).

The main TBE virus transmission season is April–November when ticks are most active because of warmer weather in the Northern Hemisphere (*12*,*110*,*112*,*133*,*134*). Peak transmission generally occurs for multiple weeks during the warm, humid summer months, typically during June*–*August in European countries. However, in central and northern Europe, two peaks might occur in summer and early fall (*12*,*15*,*16*,*23*,*37*,*111*,*112*,*115*,*135*–*137*). Unlike mosquitoborne diseases, large outbreaks of tickborne diseases do not occur. Occasional cases are reported during winter because tick activity is still possible at temperatures close to freezing (*23*,*28*,*109*,*110*,*138*).

### Clinical Manifestations and Diagnosis

#### Symptoms and Signs

Approximately three fourths of TBE virus infections are asymptomatic (*67*,*139*,*140*). Among patients who develop clinical symptoms after a bite from an infected tick, the incubation period is typically 7–14 days (range = 2–28 days) (*17*,*90*,*141*). For TBE acquired through the alimentary route, the typical incubation period is shorter, usually <2 weeks and often 2–4 days (*53*,*57*,*60*,*142*).

The most recognized clinical presentation of TBE is central nervous system infection (i.e., aseptic meningitis, meningoencephalitis, or meningoencephalomyelitis) (Box 1). Overall, meningitis is reported in approximately 35%–45% of patients, meningoencephalitis in 45%–55%, and meningoencephalomyelitis in 10% (*14*,*17*,*18*,*37*,*137*). However, younger patients (i.e., aged approximately ≤15 years) more frequently have meningitis, and the percentage of patients with more severe clinical presentations usually increases with age (*12*,*14*,*17*,*18*,*131*,*133*,*143*–*148*). Relatively mild forms of disease (e.g., undifferentiated febrile illness) can occur (*36*,*149*). A chronic form of disease has been reported from Russia linked to infection with the Siberian subtype virus and, rarely, the Far Eastern subtype virus and is possibly associated with long-term viral persistence (*18*,*36*,*150*–*153*). Progressive, slow development of neurologic symptoms often occurs, with or without an initial acute illness. In certain patients, the incubation period can be prolonged and symptoms can first manifest many years after a tick bite. A chronic relapsing form of disease also has been reported in Russia (*152*,*153*). Immunity after TBE virus infection is considered to be lifelong (*49*).

BOX 1 **Clinical manifestations, laboratory findings, investigations, and diagnostic testing for tick-borne encephalitis virus infection**

**Symptoms and signs**

- First symptoms typically manifest approximately 7–14 days (range = 2–28 days) after tick bite (or if transmission by alimentary route, often at 2–4 days).
- Acute neurologic illness (e.g., aseptic meningitis, meningoencephalitis, or meningoencephalomyelitis) or febrile illness can occur.
- Milder neurologic illness (e.g., meningitis) is more common in children (e.g., aged approximately ≤15 years) and severe illness is more common as age increases.
- Neurologic illness can be monophasic or biphasic (i.e., central nervous system manifestations occur after an initial nonspecific illness and period of remission).
- Risk factors for severe disease include older age (e.g., ≥60 years), immunocompromise, and infection with the Far Eastern subtype virus (typically found in China, Japan, Mongolia, and the eastern parts of Russia).

**Laboratory findings**

- Initial phase: leukopenia, thrombocytopenia, and elevated hepatic enzymes might be detected.
- Neurologic phase: peripheral leukocytosis, elevated erythrocyte sedimentation rate, and increased C-reactive protein.
- CSF results: pleocytosis and moderately elevated protein levels; pleocytosis is typically lymphocytic but in early disease neutrophils can predominate.

**Neuroimaging and electroencephalogram investigations**

- Sensitivity of MRI is low.
- Brain MRI changes are most commonly seen in the thalamus, often bilaterally.
- Spinal MRI can indicate T2-hyperintensities in the anterior horns of the cervical cord in patients with myelitis or radiculitis.
- Abnormal EEG findings are common and can include diffuse slowing and focal abnormalities.

**Laboratory diagnosis**

- Usually based on detection of TBE virus immunoglobulin M antibody in CSF or serum.
- Plaque reduction neutralization tests can be performed to confirm recent infection.
- Diagnostic testing can be performed at CDC. Clinicians should contact their state or local health department or the CDC Arboviral Diseases Branch, Division of Vector-Borne Diseases (970-221-6400) for assistance.

**Abbreviations:** CSF = cerebrospinal fluid; EEG = electroencephalogram; MRI = magnetic resonance imaging.

TBE can have a monophasic or biphasic illness course (i.e., an isolated neurologic illness alone or neurologic disease after an initial nonspecific illness). The monophasic disease course is the most common in infections caused by the Far Eastern and Siberian subtype viruses. The biphasic course is the most frequent in patients infected with the European subtype virus; approximately 65%–75% of patients infected with the European subtype virus have a biphasic illness course (*14*,*17*,*18*,*36*,*133*,*148*,*151*,*154*). When biphasic illness occurs, the initial phase includes nonspecific symptoms (e.g., fever, headache, malaise, myalgia, nausea, and vomiting). These symptoms usually last for a median of approximately 4 days (range = 1–10 days), followed by a period of remission of approximately 7 days (range = 1–33 days), followed by the second (neurologic) phase (*12*,*17*,*18*,*133*,*155*).

Neurologic signs and symptoms of TBE vary but can include meningeal signs, altered mental status, cognitive dysfunction (e.g., decreased concentration and memory impairment), ataxia, rigidity, tremors, and cranial nerve and limb paresis or palsies. Limb involvement is more typically unilateral than bilateral, and the upper extremities are more often affected than the lower extremities (*14*). Seizures are not common with TBE caused by the European subtype virus (*17*,*153*,*156*,*157*).

Increasing age is a key risk factor for more severe disease. Other risk factors include infection with the Far Eastern subtype virus and being immunocompromised, and certain studies have found a correlation with the monophasic illness course (*16*,*18*,*151*,*158*–*163*).

Among the limited number of published case reports of women infected during pregnancy, the clinical spectrum of illness appears similar to that of the nonpregnant population (*164*–*168*). Apart from two reports from the 1960s in which the diagnostic methods used to confirm maternal infection were unclear, all infants born to infected mothers were reported to be healthy at birth and transplacental transmission of TBE virus had not been confirmed.

#### Clinical Laboratory Findings

Clinical laboratory findings with TBE are nonspecific. In the initial phase of a biphasic illness, findings can include leukopenia, thrombocytopenia, or elevated hepatic enzymes (*145*,*149*,*154*). In the neurologic phase of disease, findings can include a peripheral leukocytosis, an elevated erythrocyte sedimentation rate, and increased C-reactive protein levels (*12*,*17*,*37*,*133*,*146*). Cerebrospinal fluid (CSF) testing usually indicates a pleocytosis, typically lymphocytic, with moderately elevated protein levels (*12*,*17*,*37*,*131*,*133*). However, early in disease, neutrophils can predominate in CSF.

#### Neuroimaging and Electroencephalogram Investigations

Magnetic resonance imaging (MRI) occasionally detects abnormalities in the brain or spinal cord; however, sensitivity is low for diagnosis of TBE (*169*). In one prospective study in Germany, 18% (18 of 102) of patients with MRI results had abnormal findings, and in a retrospective study from Austria, 9% (four of 45) of patients with MRI results had abnormalities considered TBE related (*17*,*170*). Changes, when present, are most commonly observed in the thalamus, often bilaterally, and less often in the cerebellum, basal ganglia, brainstem, or other locations (*17*,*147*,*171*–*174*). In patients with myelitis, radiculitis, or both, either alone or in association with encephalitis, spinal MRI can indicate changes such as T2-hyperintensities in the anterior horns of the cervical cord (*171*,*175*–*179*). Computerized tomography scans do not usually identify any abnormalities (*169*). Abnormal electroencephalogram findings are common and can include diffuse slowing and focal abnormalities (*17*,*132*,*133*,*147*,*173*).

#### Laboratory Diagnosis

The laboratory diagnosis of TBE usually is based on detection of virus-specific immunoglobulin M (IgM) antibody in CSF or serum (*180*). An IgM enzyme-linked immunosorbent assay is routinely used for testing samples and usually is positive when neurologic symptoms are present. However, cross-reactivity with other flavivirus antibodies can occur because TBE virus shares common antigenic sites within its E protein with multiple other flaviviruses (*49*). Plaque reduction neutralization tests can be performed to discriminate between cross-reacting antibodies attributable to another primary flavivirus infection or to confirm recent TBE virus infection on the basis of a fourfold or higher increase in virus-specific neutralizing antibodies between acute- and convalescent-phase serum specimens. However, in patients who have been infected previously by another flavivirus or vaccinated with a different flavivirus vaccine (e.g., Japanese encephalitis or yellow fever vaccine), cross-reactive antibodies can make identifying a specific etiologic agent difficult (*180*). Vaccination history, date of symptom onset, and information about other flaviviruses known to circulate in the geographic area that might cross-react in serologic assays should be considered when interpreting results. In addition, possible antibody persistence from a previous TBE virus infection should be considered; serum IgM antibodies typically are detectable for approximately 3–4 months after infection but can persist for ≥3 years (*12*,*181*,*182*).

TBE virus occasionally has been isolated, or TBE viral RNA has been detected by nucleic acid amplification tests (NAATs), in serum, whole blood, urine, or CSF samples when a patient has neurologic illness (*50*,*51*,*67*,*92*,*127*,*168*,*183*–*187*). Although these methods are insufficiently sensitive for routine diagnostic purposes, NAATs can be of value in patients who are immunocompromised (*158*,*188*,*189*). In addition, if testing is done during the initial febrile (viremic) phase of illness before neurologic symptoms develop and antibodies are measurable, RNA often can be detected; however, patients usually only are tested after neurologic disease manifests (*49*,*169*,*184*). In fatal encephalitis cases, TBE virus RNA has been detected in brain tissue (*68*,*184*).

No commercially available tests for TBE virus infection are available in the United States. Diagnostic testing can be performed at CDC. Clinicians should contact their state or local health department or the Arboviral Diseases Branch, Division of Vector-Borne Diseases (970-221-6400) for assistance with diagnostic testing.

#### Treatment and Management

No specific antiviral treatment for TBE is available. Patient management consists of supportive care, treatment of symptoms, and interventions to prevent secondary complications (e.g., aspiration pneumonia or urinary tract infection) (*153*,*169*). Patients with meningoencephalitis should be closely observed because coma or neuromuscular paralysis leading to respiratory failure can develop rapidly (*36*).

An anti-TBE virus intravenous immunoglobulin (IVIG) preparation was previously used in Europe for postexposure prophylaxis or treatment. However, no effectiveness data from controlled clinical trials are available, and IVIG use was discontinued after reports of suspected antibody-dependent enhancement of infection resulting in a more severe course of disease (*190*–*193*). In Russia and Kazakhstan, specific anti-TBE virus IVIG preparations continue to be used; information on their effectiveness is published primarily in the non-English literature (*153*).

#### Outcome and Sequelae

The outcome of TBE largely depends on the patient’s age, clinical form of the disease, and virus subtype (*194*). Among patients with neurologic disease and infected with the European subtype virus, the case fatality rate is usually <2% (*14*,*17*,*18*,*37*,*86*,*110*,*111*,*114*,*146*,*195*,*196*). Fatality rates from infection with the Siberian subtype virus are higher but rarely exceed 6%–8% (*151*). Rates of 20%–40% were historically described with the Far Eastern subtype virus, although the extent of study methodology and patient inclusion criteria as contributing factors is unclear (*62*,*151*,*197*). In China, where the Far Eastern subtype virus is found, case-fatality rates were >25% in the 1950s; however, rates of <10% have been reported since the 1980s, purportedly related to improved disease awareness and quality of medical care (*151*,*198*). In Russia, where the Far Eastern and Siberian subtype viruses predominate, TBE mortality rates of approximately 2% have recently been reported (*153*).

Studies to assess frequency of sequelae have used variable symptom definitions, types of cohorts, investigation approaches, and durations of follow-up after illness to measure outcomes, making interpretation and comparison of studies difficult. A limitation of multiple studies is incomplete follow-up among the persons in the cohort, potentially biasing results. Among patients infected with the European subtype virus, sequelae have been reported in 20%–40% overall, including neurologic sequelae (e.g., limb paresis or paralysis) in up to 10% (*17*,*18*,*155*,*194*,*196*). Sequelae have been reported with higher frequency after infection with the Far Eastern and Siberian subtype viruses, but the reported differences might be a result of methodologic differences in published reports.

The severity of reported sequelae ranges from mild symptoms with limited to no effect on quality of life to severe sequelae that interfere with activities of daily living. Reported serious outcomes of infection include permanent limb or cranial nerve palsies or paralysis, ataxia, and dysphasia (*155*,*194*). Milder symptoms include cognitive impairment (e.g., difficulties with memory or concentration), headaches, fatigue, tremors, hearing loss, emotional lability, or minor problems with balance or coordination (*17*,*18*,*37*,*196*). In a case-control study in Sweden with 92 patients and 58 controls with follow-up conducted from 2 to 15 years (median = 5.5 years) after TBE virus infection, patients scored significantly lower than controls in the domains of memory and learning, executive function (i.e., initiative and motivation), vigilance (i.e., concentration, attention, and fatigue), and physical impairment (i.e., fine motor skills, coordination, and balance) (*199*).

Certain symptoms can improve or resolve during the weeks to months after hospitalization (*200*). In one study, the median time to recovery was 13 weeks (range = 2–156 weeks) among the patients who recovered completely or had only unrelated ongoing health issues (63%; 72 of 114) (*196*). However, patients occasionally have worsening of sequelae over time (*18*,*173*).

Severe outcomes are more frequent with increasing age and might be of particular concern for persons aged approximately ≥60 years (*201*). Older age has been correlated with a longer duration of hospitalization, lengthier time to recovery, higher case-fatality rate, and increased risk for sequelae (*17*,*86*,*111*,*131*,*194*,*196*). The association between older age and poor outcomes is likely related to immunosenescence with increasing age (*202*). Among children, deaths are rare and neurologic sequelae occur at low rates (<3%) (*12*,*14*,*129*,*133*,*148*,*203*). However, permanent, severe neurologic deficits can occur and subtle deficits (e.g., cognitive problems, headache, fatigue, or irritability) might be common (*19*,*127*,*173*,*204*).

### TBE Among Travelers

TBE is rare among U.S. travelers to areas where the disease is endemic. Twelve TBE cases have been reported among U.S. adult and pediatric civilian (i.e., nonmilitary) travelers, including one case in 1979 and 11 cases during 2001–2021 (*180*,*205*,*206*) (Table 1). During 2001–2021, the mean was <1 reported case (range = 0–2 cases) annually. However, TBE cases might not have been identified if the illness was diagnosed overseas or if a clinician did not consider TBE in the differential diagnosis for a returning traveler with a compatible illness. On the basis of approximately 20–25 million U.S. citizen trips to countries with TBE risk each year, and a mean of <1 diagnosed TBE case each year, the overall incidence of TBE among U.S. civilian travelers is low (*207*). However, certain persons who travel abroad will be at increased risk for infection because of location and season of travel, their activities, and other factors (Box 2).

**TABLE 1:** Tick-borne encephalitis cases among U.S. civilian travelers, 1979–2021*

Case no.	Year	Age group, yrs	Sex	Location of potential exposure to TBE virus	Month of onset	Duration of travel, days^†^	Reported activities with risk for tick exposure	Outcome
1	1979	0–9	F	Hungary	NA	NA	NA	Recovered
2	2001	50–59	M	Russia	June	10	Fishing	Mild cognitive impairment
3	2004	20–29	M	Russia	August	45	NA	Recovered
4	2006	40–49	M	Sweden	July	18	NA	Recovered
5	2007	10–19	F	China	July	23	Hiking	Severe neurologic sequelae
6	2008	10–19	M	Czech Republic	August	69	Extensive outdoor exposure in rural areas	Recovered
7	2012	30–39	M	Czech Republic	August	28	Hiking	Recovered, short-term sequelae
8	2012	40–49	M	Finland	August	16	Camping	Recovered, unknown if sequelae
9	2013	70–79	M	Russia (including Siberia)	July	24	Hiking, picnicking, rural homestay	Unknown, discharged from hospital to rehabilitation facility
10	2017	40–49	M	Switzerland	August	7	NA	Recovered, mild sequelae
11	2018	60–69	M	Sweden	May	16	Trail running, manual work outdoors	Recovered, initial sequelae resolved after several months
12	2019	0–9	M	Austria, Switzerland	June	10	Hiking	Recovered

**Abbreviations:** F = female; M = male; NA = not available; TBE = tick-borne encephalitis.

* Excludes U.S. military personnel and their dependents.

^†^ Approximate number of days spent in areas where TBE is endemic.

BOX 2 **Factors that increase risk for tick-borne encephalitis among U.S. travelers to areas where the disease is endemic**

**Season**

- TBE virus transmission is primarily during the warmer months of April–November when ticks are most active in the Northern Hemisphere.

**Location**

- TBE virus transmission occurs within focal areas of countries in the geographic region extending from western and northern Europe through to northern and eastern Asia.
- Information on countries with TBE risk areas is available on the CDC website (https://www.cdc.gov/tick-borne-encephalitis/geographic-distribution/index.html). The information should be interpreted cautiously because TBE virus transmission can be highly variable within risk areas and from year to year.
- Ticks typically are found in woodland habitats including in coniferous or deciduous forests and on the forest edges in the transition zone between forests and grasslands.

**Activities and occupations**

- Certain recreational activities can increase the risk for exposure to ticks (e.g., hiking, camping, cycling in woodland areas, hunting, fishing, birdwatching, and collecting mushrooms or berries).
- Occupational risk might exist for persons with exposure to ticks (e.g., farmers, forestry workers, military personnel, or researchers conducting field work in rural areas).
- Infection can follow ingestion of unpasteurized milk and milk products from infected goats, sheep, or cattle.

**Duration**

- Activities undertaken are more important than time spent abroad. Among U.S. travelers, TBE cases have occurred after travel as short as 7–10 days; however, a longer duration of travel, residence, or repeated travel to areas of where the disease is endemic might increase the likelihood of exposure to TBE virus.

**Other considerations**

- Risk for severe disease increases with age, and poor outcomes might be of particular concern for persons aged ≥60 years.
- Persons with altered immunocompetence can have severe TBE and have a higher risk for a fatal outcome; however, immunocompromise and immunosuppression are precautions for vaccination.

**Abbreviation:** TBE = tick-borne encephalitis.

Among the 12 TBE cases diagnosed in U.S. civilian travelers during 1979–2021, a total of 10 (83%) occurred in males, the median age was 38 years (range = 4–79 years), and infections were acquired in Europe, Russia, or China. Travel, and thus exposure to TBE virus, for all patients occurred during May–August. Among 11 travelers for whom information was available on duration of travel in areas where TBE is endemic, the median travel duration was 18 days (range = 7–69 days). All eight travelers with available data reported activities with risk for tick exposure, including hiking, camping, fishing, and trail running. Clinical illness occurred in a biphasic manner in eight (67%) patients. Eight (67%) patients had meningoencephalitis and four (33%) had meningitis, and no deaths occurred. Seven (58%) patients recovered completely, two (17%) had mild cognitive sequelae, one (8%) recovered but information on possible sequelae was unavailable, one (8%) had severe neurologic sequelae including dysarthria and mild limb bradykinesia, and one (8%) was discharged from acute care to a rehabilitation facility but their clinical outcome was unknown.

In addition to the 12 cases among civilian travelers, 12 TBE cases were diagnosed among U.S. military personnel (n = 8) or their dependent children (n = 4) during 2012–2021 (*208*–*210*) (Table 2). One case occurred in 2012, and the remaining 11 occurred during 2017–2021. At the time of infection, all persons were living in Germany; nine persons had specific information available and all were living in Baden-Württemberg or Bavaria, Germany’s two states with the highest number of reported annual TBE cases (*86*). On the basis of a mean of 1.2 cases per year among approximately 50,000 U.S. military personnel and dependents living in Germany, the TBE risk was similar to that of the local population of Baden-Württemberg and Bavaria where annual TBE incidence during 2012–2018 ranged from 0.7 to 2.0 cases per 100,000 population (*86*). Among the 12 TBE cases, 10 (83%) were in males, the median age was 33 years (range = 2–47 years), illness onsets occurred during April–November, and nine (75%) had neurologic illness. Five (42%) patients recovered, including two who had short-term sequelae before complete recovery; four (33%) had no outcome information reported; and three (25%) experienced moderate sequelae.

**TABLE 2:** Tick-borne encephalitis cases among U.S. military personnel and their dependents, 2012–2021

Case no.	Year	Age group, yrs	Sex	State of residence, Germany	Month of onset	Outcome
1	2012	30–39	M	Baden-Württemberg	September	Recovered
2	2017	40–49	M	Bavaria	June	Moderate sequelae
3	2017	30–39	M	Bavaria	July	Recovered
4	2017	30–39	M	Bavaria	July	Recovered, short-term sequelae
5	2017	30–39	M	Bavaria	November	Moderate sequelae
6	2018	0–9	M	Baden-Württemberg	April	Recovered
7	2018	10–19	M	Baden-Württemberg	June	Recovered, short-term sequelae
8	2018	0–9	F	Baden-Württemberg	September	Moderate sequelae
9	2019	40–49	M	NA	June or July	NA
10	2020	30–39	M	Bavaria	May, June, or July	NA
11	2021	0–9	F	NA	June or July	NA
12	2021	30–39	M	NA	June or July	NA

**Abbreviations:** F = female; M = male; NA = not available; TBE = tick-borne encephalitis.

In Europe, a median of 36 traveler cases (range = 25–65 cases) wase reported to the European Centre for Disease Prevention and Control each year during 2014–2020 among the approximately 2,000–3,800 TBE cases reported annually (*211*). However, because TBE is endemic in multiple areas of Europe and TBE vaccines are available, the number of traveler cases prevented by vaccination is unknown (*212*). Most cases occurred among persons who reported undertaking activities with risk for tick exposure, with only rare case reports of travelers with TBE acquired through ingestion of unpasteurized dairy products (*142*,*213*–*216*). Local population TBE incidence data for multiple countries where the disease is endemic in Europe are published annually by the European Centre for Disease Prevention and Control; however, infection risk for a traveler cannot be inferred from these data because the data might be influenced by surveillance methods, reporting practices, and vaccination coverage (*104*).

### TBE Among Laboratory Workers

At least 47 laboratory-acquired TBE virus infections have been reported globally, and approximately all occurred before 1980 (*63*–*65*,*67*). Among these 47 infections, 37 (79%) resulted in disease, and the remainder were asymptomatic infections. At least four of the infections occurred among U.S. laboratory workers; three cases were overt disease with two deaths reported, one was an asymptomatic infection, and all occurred before 1980. None of the infected laboratory workers was known to have received TBE vaccine. Limited information was available on transmission routes; however, all 10 cases with information reported were attributed to aerosolization during laboratory procedures or handling of infected animal waste. Transmission through accidental percutaneous or mucosal exposures is possible. Work with TBE virus typically is restricted to biosafety level (BSL)-4 facilities and practices (*217*).

## Methods

The ACIP TBE Vaccine Work Group was formed in September 2020 to 1) review information on the epidemiology, clinical presentation, diagnosis, treatment, and outcome of TBE; 2) review data on disease risk and burden for travelers and laboratory workers; 3) review data on TBE vaccine safety, immunogenicity, and effectiveness; and 4) draft evidence-based vaccination recommendations for ACIP’s review. The work group included two ACIP members, one ex officio member from each of the National Institutes of Health and the FDA, liaison representatives from the American Academy of Pediatrics and the International Society of Travel Medicine, and technical advisors. The expertise represented by these members included infectious diseases, pediatrics, travel medicine, public health, arbovirology, entomology, vaccinology, and vaccine policy. The work group met via conference call 27 times during September 2020–March 2022 with participation from CDC consultants and staff members from various CDC divisions.

The Grading of Recommendations Assessment, Development, and Evaluation (GRADE) approach was used to review and evaluate available data (*218*). Details on the methods used, including the policy question developed, ranking of outcomes, systematic review protocol, inclusion criteria, evidence summary, and certainty assessment are provided in GRADE for TBE vaccine (*219*). In developing the TBE vaccine recommendations, the work group also assessed additional factors as outlined in the ACIP Evidence to Recommendations (EtR) framework, including the public health importance of TBE, population values, stakeholder acceptability, resource use considerations, health equity issues, and feasibility of implementation (*220*). Detailed information is available in the ACIP EtR framework for TBE vaccination for persons who travel abroad and ACIP EtR framework for TBE vaccination for laboratory workers (*221*,*222*).

Work group members or the manufacturer gave presentations on vaccine immunogenicity, safety, and other topics related to the development of the TBE vaccine recommendations at ACIP meetings during October 2020–January 2022. The work group presented the TBE vaccine EtR for persons who travel abroad and for laboratory workers and preliminary vaccination recommendations to ACIP during its January 12, 2022, meeting. ACIP voting members unanimously approved the TBE vaccine recommendations for persons who travel abroad and for laboratory workers at the February 23–24, 2022, ACIP meeting (*223*).

## Summary of Findings on TBE Vaccine

### Manufacture and Licensure

On August 13, 2021, FDA approved a TBE vaccine manufactured by Pfizer, Inc. for use in persons aged ≥1 year (*224*). The vaccine is licensed in the United States under the trade name Ticovac; in Europe, it is also marketed as FSME-Immun (*3*). The vaccine is an inactivated, whole virus vaccine, prepared using a European subtype TBE virus. The vaccine has an adult (0.5 mL) formulation for use in persons aged ≥16 years and a pediatric (0.25 mL) formulation for use in children aged 1–15 years. In both age groups, the schedule includes a 3-dose primary series; a booster can be administered ≥3 years after completion of the primary series if ongoing exposure or re-exposure to TBE virus is expected. Pfizer’s TBE vaccine is the only U.S.-licensed TBE vaccine; other TBE vaccines are manufactured in China, Europe, and Russia but are not available in the United States (*153*).

An earlier formulation of Pfizer’s TBE vaccine was first licensed in Austria in 1976 (*2*). Changes to the vaccine formulation over time have included removal of thimerosal and a transition of the production virus seed from mouse brain suspension to chick embryo fibroblast cells. The current adult and pediatric formulations became available in Europe in 2001 and 2003, respectively (*3*).

### Vaccine Protection for TBE

#### Measurement of Protection

No randomized controlled trials (RCTs) to demonstrate the efficacy of TBE vaccine for prevention of clinical disease have been conducted; the low incidence of TBE in areas where the disease is endemic would make such trials infeasible. Vaccine effectiveness (VE) studies for TBE vaccine using a different schedule, with a previous vaccine formulation or with VE assessed in combination with another TBE vaccine or both, have been published (*126*,*130*,*225*–*230*); however, no studies assessing VE of the TBE vaccine alone in its current formulation and according to the U.S. licensed schedule exist. Clinical trials have been conducted using immunogenicity endpoints. TBE virus neutralizing antibodies are believed to confer protection against disease on the basis of animal and human studies (*231*,*232*). A neutralizing antibody titer of ≥10 is considered a seropositive result and is generally used to indicate protection. However, a correlate of protection has not been formally established, and no standardized reference reagents are available (*232*).

#### Level of Cross-Protection for TBE Virus Subtypes and Related Viruses from the TBE Vaccine

In addition to the genetic and antigenic similarity of the three TBE subtype viruses, data from human and animal studies suggest that cross-protection for heterologous subtype viruses from the European subtype-based TBE vaccine is likely. However, data are limited, and VE has not been demonstrated.

In one comparative sequence analysis, phylogenetic relations were determined for 25 TBE virus strains from the three genetic lineages (*6*). Variability among strains in the envelope protein, the main target for neutralizing antibodies at the amino acid level, was ≤2% within and 4%–6% among subtype viruses. In older human studies using the previous vaccine formulation, neutralizing antibody titers were similar against various European, Far Eastern, and Siberian TBE virus strains; in animal studies, generally good protection was demonstrated for vaccinated mice challenged with strains from the three TBE virus subtypes (*233*–*235*). In a more recent study, 41 adults were vaccinated with the current formulation of the TBE vaccine, and the ability of postvaccination sera to neutralize representative European (n = 2), Far Eastern (n = 2), and Siberian (n = 1) subtype TBE pseudoviruses (created using a West Nile virus backbone and encoding the membrane and envelope proteins of the various TBE virus strains) was investigated. The sera successfully neutralized all the pseudoviruses, with no statistically significant differences between mean neutralizing antibody titers (*236*). In a mouse study, no statistically significant differences were found in mean neutralizing antibody titers against European and Far Eastern TBE pseudoviruses (*237*). In addition, the mice were protected against challenge with both strains in a dose-dependent manner, and the 50% protective dose values were not significantly different. In another mouse study, postvaccination neutralizing antibody titers measured using Far Eastern (n = 1), European (n = 1), and Siberian (n = 1) TBE virus strains were similar (*238*). Ten mice vaccinated with various dilutions of the TBE vaccine also underwent viral challenge with a recent Siberian subtype virus isolated in 2010. Although the percentage of lethal outcomes varied, the minimum vaccine dose protecting 50% of the mice was considered to be within acceptable limits.

Data are limited on potential cross-protection of TBE vaccine for Powassan virus infection. One study demonstrated limited neutralization of Powassan virus by sera from persons vaccinated with a European subtype virus TBE vaccine (*239*). A study of mice vaccinated with a Far Eastern subtype virus TBE vaccine demonstrated minimal to no neutralization of Powassan virus by the mice sera, and the mice were not protected against a lethal Powassan virus challenge (*240*). These data suggest minimal to no cross-protection against Powassan virus afforded by TBE vaccination.

#### Vaccine Effectiveness

VE studies have been conducted in four countries in Europe (Austria, Germany, Latvia, and Switzerland) where both the U.S.-licensed vaccine and another non–U.S.-licensed European subtype vaccine are in use (*126*,*130*,*225*–*230*). The European subtype is the only TBE virus circulating in three of these countries; in Latvia, all three virus subtypes circulate with seeming predominance of the European subtype virus (*241*,*242*). No studies have investigated VE for the Far Eastern or Siberian subtype viruses. The studies all have one or more limitations, particularly when considering VE for the U.S.-licensed vaccine (e.g., inclusion of persons who received an older formulation of the vaccine, use of different dosing schedules to the U.S. schedule [e.g., >1 booster dose], unknown or potentially inaccurate estimates of market share for each vaccine, limited data by age group including from persons aged >50 years, or weaknesses in methods used to estimate vaccination coverage) (*243*).

Overall, VE estimates for the European subtype virus TBE vaccines against disease caused by the European subtype virus after ≥3 doses of vaccine are 91%–99% for prevention of various outcomes (e.g., all TBE disease and hospitalization) (*225*–*229*). One study in Germany covering the period 2018–2020 used a case-control study design to estimate VE specifically for the U.S.-licensed TBE vaccine; VE was 93% (95% CI = 87%–97%) for prevention of neurologic or nonneurologic disease after ≥3 doses in adults and children; however, the percentage of persons who received only 3 primary doses and 1 booster dose (i.e., in accordance with the U.S.-licensed schedule) was unavailable (*228*). In a study that included data from southern Germany and Latvia, VE estimates for prevention of neurologic or nonneurologic TBE after 3 doses when the interval after the third dose was <3 years were 95% (95% CI = 93%–97%) and 99% (95% CI = 98%–100%), respectively (*226*). Estimates also were available by age group; in Germany and Latvia, VE estimates were 94% (95% CI = 83%–98%) and 98% (95% CI = 87%–100%) for persons aged ≤17 years, 97% (95% CI = 95%–98%) and 99% (95% CI = 97%–100%) for persons aged 18–59 years, and 88% (95% CI = 80%–93%) and 100% for persons aged ≥60 years, respectively. A study conducted in Austria demonstrated that VE to prevent hospitalization among patients aged ≥1 year after ≥1 dose was 95% (95% CI = 95%–96%) regardless of whether vaccinations were administered according to schedule (*130*). VE was slightly lower to prevent severe disease compared with mild disease, with estimates of 95% (95% CI = 94%–96%) and 97% (95% CI = 96%–98%), respectively. The lower VE was most evident for children aged 1–16 years, with estimates of 83% (95% CI = 70%–90%) for severe disease compared with 95% (95% CI = 91%–97%) for mild disease.

#### Immunologic Response After a 3-Dose Primary Series in Adults and Children

The pivotal adult immunogenicity study with the current formulation of the vaccine was conducted in Poland among adults aged 16–64 years (*224*,*244*). Among 416 adults seronegative at baseline, 411 (99%) were seropositive at 21–28 days after dose 3 of the primary series with a geometric mean titer (GMT) of 259 (95% CI = 235–285). In a follow-up study, the seropositivity rate at 2 years after dose 3 was 96% (242 of 252) and at 3 years after dose 3, immediately before the booster dose, was 94% (229 of 243) (*245*,*246*). Although the seropositivity rates at 2 and 3 years were high, GMTs had declined to 62 (95% CI = 53–71) at 2 years and 49 (95% CI = 42–56) at 3 years after completion of the primary series.

Key pediatric data came from a study conducted in Austria, Germany, and Poland among children aged 1–15 years (*245*,*247*). At 35–42 days after dose 3 of the primary series, 358 (99%) of 360 children were seropositive and GMT was 382 (95% CI = 351–415). Seropositivity rates were ≥99% in all age groups, but GMT was significantly lower among children aged 7–15 years (307 [95% CI = 272–347]) than among children aged 3–6 years (469 [95% CI = 399–552]) and 1–2 years (568 [95% CI = 527–612). In a follow-up study, seropositivity rates at 2 and 3 years after completion of the 3-dose primary series for all age groups combined were 98% at both time points (i.e., 352 of 358 children seropositive at year 2 and 346 of 353 at year 3; intent-to-treat data set) (*245*,*248*). When comparing GMTs by age group at these later time points, they were again lower in the group aged 7–15 years compared with younger age groups. GMTs for the children aged 7–15 years, 3–6 years, and 1–2 years at 2 years were 111 (95% CI = 95–129), 204 (95% CI = 165–252), and 154 (95% CI = 125–189), respectively, and at 3 years were 97 (95% CI = 83–113), 188 (95% CI = 150–236), and 166 (95% CI = 135–205), respectively. GMTs among children demonstrated a moderate decline between 1 month and 2 years after the primary series, then remained relatively stable between 2 and 3 years.

#### Immunologic Response After a 3-Dose Primary Series in Older Adults

Data are limited on the immunologic response measured by neutralizing antibody titer to the primary series of TBE vaccine among older adults, particularly those aged ≥65 years. A study conducted in Poland compared seropositivity rates among adults aged 16–49 years (n = 170) and 50–79 years (n = 170, including 31 adults aged ≥65 years) (*3*,*245*). The seropositivity rates at 21 days after dose 3 among younger and older age groups were 100% (144 of 144 [95% CI = 98%–100%]) and 99% (151 of 153 [95% CI = 95%–100%]), respectively. However, GMT among persons aged 16–49 years (208 [95% CI = 174–247]) was significantly higher than GMT among adults aged 50–79 years (104 [95% CI = 86–127]). A second study investigated the immune response in healthy, initially seronegative, older adults in Switzerland; all were aged ≥70 years but an age range was not provided (*249*). At 4 weeks after dose 3, the seropositivity rate was 99% (136 of 137) and GMT was 71 (*249*). In the pivotal adult immunogenicity study conducted in Poland, a post hoc analysis compared seropositivity rates and GMTs among younger persons (aged 18–50 years [n = 211]) and older persons (aged 51–67 years [n = 41, including 15 aged >60 years]) (*245*,*246*). At 21–28 days after dose 3 of the primary series, seropositivity rates were 100% in both groups but GMT was significantly higher for the younger age group (303 [95% CI = 269–341] versus 122 [95% CI = 87–173). At 3 years after dose 3, the seropositivity rate in the younger age group was significantly higher (97% [95% CI = 93%–99%]) than in the older age group (83% [95% CI = 68%–93%]), and GMT also was significantly higher (55 [95% CI = 47–64] versus 26 [95% CI = 18–36]).

Overall, seropositivity rates immediately after completion of the 3-dose primary series are high among older persons but levels of neutralizing antibodies are lower than among younger adults. Over time postvaccination, lower neutralizing antibody levels continue to be observed among older adults and they are more likely to become seronegative.

#### Immunologic Response After a Booster Dose in Adults and Children

The immunologic response to a booster dose administered 3 years after the 3-dose primary series was investigated in adults aged 18–67 years at the time of the booster dose (*245*,*246*). At 21–35 days after a booster dose, all 240 persons were seropositive. GMT increased ninefold from the prebooster titer of 49 (95% CI = 42–56) to the postbooster titer of 428 (95% CI = 394–464). In a follow-up study, regular blood draws were conducted to investigate the neutralizing antibody response for 10 years after the booster dose. The seropositivity rate at 5 years after a booster dose was 94% (209 of 222) and at 10 years was 85% (189 of 222). GMT decreased to 99 (95% CI = 87–112) at 2 years postbooster, then slowly declined to 32 (95% CI = 24–43) at the 10-year time point (*250*,*251*).

A pediatric booster dose study was conducted among children who had been enrolled in a vaccine immunogenicity study at ages 1–15 years; at the time of the booster study 3 years later most received the pediatric booster dose (0.25 mL) but approximately 21% were aged 16–18 years and received the adult dose (0.5 mL) for the booster (*248*). At 21–35 days after a booster, the seropositivity rate was 100% (172 of 172), and GMT increased sixfold from 59 (95% CI = 51–68) prebooster to 359 (95% CI = 316–409) postbooster. In a 10-year follow-up study, neutralizing antibody levels were measured among children who had received a booster dose 3, 4, or 5 years after the 3-dose primary series; approximately 85%, 14%, and <1% of the full cohort received the booster dose at these respective intervals (*248*). Seropositivity rates at 5 years and 10 years after a booster dose administered 3–5 years after the primary series were 99% (155 of 156) and 90% (140 of 155), respectively. GMTs demonstrated an initial moderate decrease from 381 approximately 1 month postbooster to 162 at 3 years postbooster, with a subsequent gradual decline to 54 at 10 years postbooster. Data on seropositivity rates and GMTs were not provided separately for the groups with boosters administered at 3, 4, or 5 years. At 10 years after the booster, the proportions seropositive by age group were 86% (25 of 29), 92% (23 of 25), 93% (57 of 61), and 88% (35 of 40) for children who had begun the primary series at age 1–2 years, 3–6 years, 7–11 years, and 12–15 years, respectively; none of these differences were statistically significant. GMTs at 10 years postbooster for these age group were 34, 80, 66, and 43, respectively; for each age group, the lower bound of the 95% CI for GMT was >10, and differences between GMTs were not statistically significant (Pfizer, Philadelphia, PA, unpublished data, 2021).

#### Immunologic Response After a Booster Dose in Older Adults

Data are limited on the immunologic response measured by neutralizing antibody titer to a booster dose among older adults. At 21–35 days after a booster dose administered 3 years after the 3-dose primary series to 41 adults aged 51–67 years, 100% were seropositive (*245*,*246*). GMT increased twelvefold from the prebooster titer of 26 (95% CI = 18–36) to the postbooster titer of 303 (95% CI = 228–403). GMTs in 199 adults aged 18–50 years were significantly higher prebooster (55 [95% CI = 47–64]) and postbooster (459 [95% CI = 424–497]).

No data are available for older adults on the longer-term immunologic response measured by neutralizing antibody titer after a booster dose of the current formulation of the TBE vaccine. Limited data are available from a booster dose study in which the primary series for approximately 75% of adults was a 3-dose series of the U.S.-licensed TBE vaccine and for 25% was 2 doses of another European TBE vaccine and 1 dose of the U.S.-licensed TBE vaccine; all persons received a booster dose with the U.S.-licensed TBE vaccine (*250*). Overall, 54 adults were aged 50–60 years and 10 were aged 61–70 years at the time of the booster dose. Seropositivity rates 5 years after a booster dose were 92% (47 of 51) for adults aged 50–60 years and 75% (six of eight) for adults aged 61–70 years; GMTs were 76 (95% CI = 53–110) and 24 (95% CI = 5–121), respectively. At 10 years, seropositivity rates were 75% (38 of 51) and 38% (three of eight), and GMTs were 26 (95% CI = 13–50) and 1 (95% CI = 0.1–20).

The limited sample sizes make interpretation of these data difficult. Results from additional studies on immunogenicity among older adults, although not directly relevant to the U.S-licensed TBE vaccine, have suggested a reduced immunologic response or steeper decline in neutralizing antibodies among older adults after a booster dose, likely related to immunosenescence (*202*,*252*–*254*). However, in the absence of measurable neutralizing antibodies, vaccinated persons can usually still mount a rapid anamnestic response to infection, and T-cells also likely play a key part in the response. As a result, low neutralizing antibody levels might not necessarily indicate lack of protection (*254*,*255*).

#### Immunologic Response After an Incomplete Primary Series (1 or 2 Doses) in Adults and Children

Data on the short-term immunologic response after 1 dose of TBE vaccine were available from a study investigating kinetics of the immune response among adults when the first 2 vaccine doses were administered 10–14 days apart (*3*,*245*). In this study conducted in Poland, the seropositivity rate at approximately 12 days after dose 1 among persons aged 16–49 years was 52% (79 of 153) and among adults aged 50–79 years was 27% (43 of 159). GMTs for the younger and older groups were 11 (95% CI = 10–13) and eight (95% CI = 7–10), respectively.

Multiple studies among adults investigated the immunologic response after 2 doses of TBE vaccine. The seropositivity rates among healthy adults at approximately 3–4 weeks after dose 2 ranged from 83% to 100% in six studies (*3*,*245*,*249*,*256*–*259*). Seropositivity rates were 72% and 87% at 5 months and 27% at 11 months on the basis of three studies that provided results at one of these time points (*3*,*244*,*245*,*259*). One of these studies reported a lower seropositivity rate among adults aged 50–79 years (65%) than adults aged 16–49 years (79%) (*3*,*245*). In studies among adults that measured GMTs immediately before and 3–4 weeks after dose 3 of the primary series, GMTs were close to 10 before dose 3 but administration of the final dose of the primary series resulted in a greater than tenfold increase in GMT (*3*,*244*,*245*,*256*).

Studies among pediatric populations also measured the immunologic response after 2 doses of TBE vaccine, and results were similar to those of studies among adults. Among healthy children and adolescents, seropositivity rates at 3–5 weeks after dose 2 ranged from 84% to 100% in three studies (*245*,*247*,*260*). Rates were 95% at 5 months and 38% at 9 months on the basis of two studies that reported results at one of these time points (*245*,*261*,*262*).

Estimates of VE after 2 doses of vaccine have been published, with similar limitations to those that assessed VE after ≥3 doses (i.e., no studies of VE for the Far Eastern or Siberian subtype viruses or for the U.S.-licensed vaccine alone, inclusion of persons vaccinated with an older vaccine formulation, and uncertainties with vaccine coverage estimates). VE estimates against TBE caused by the European subtype virus in studies with the European vaccines were 83%–99% overall after 2 doses of vaccine when the interval after dose 2 was ≤12 months (*225*–*228*). In recent studies in southern Germany and Latvia, estimates by age group in each location were 94% (95% CI = 84%–98%) and 92% (95% CI = 74%–97%), respectively, for persons aged ≤17 years, 97% (95% CI = 94%–99%) and 98% (95% CI = 97%–99%) for those aged 18–59 years, and 99% (95% CI = 92%–100%) and 100% for those aged ≥60 years (*226*). If the interval after dose 2 was >12 months, VE was lower; the lowest VE estimate was 81% (95% CI = 39%–94%) among children aged ≤17 years in Latvia (*226*).

#### VE for Alimentary TBE

VE against TBE acquired through the alimentary route has been estimated on the basis of a 2017 TBE outbreak investigation in Germany that was identified after a patient with a history of consumption of raw goat milk was hospitalized with meningoencephalitis (*263*). Among 20 persons who had consumed the raw milk and were included in the investigation, 65% (n = 13) had laboratory-confirmed TBE presenting as nonspecific febrile illness. Among vaccinated persons, the attack rate was 17% (one of six); the infected person’s last TBE vaccine dose had been >15 years previously. Among unvaccinated persons, the attack rate was 86% (12 of 14). Although the number of included persons was limited, the estimated VE was 81% on the basis of these attack rates.

#### Concomitant Administration of TBE Vaccine with Other Vaccines

Data are limited on co-administration of TBE vaccine with other vaccines. One open label, nonrandomized clinical trial investigated concomitant administration of TBE vaccine and yellow fever vaccine (Stamaril) among adults aged 18–55 years (*264*). Among persons who received TBE and yellow fever vaccines concomitantly (n = 38), TBE vaccine alone (n = 19) or yellow fever vaccine alone (n = 20), no significant differences were identified between groups in TBE neutralizing antibody titers at 30 days after dose 3 of TBE vaccine, or in final yellow fever neutralizing antibody titers (taken at day 210 in the concomitant group and day 60 in the yellow fever vaccine alone group).

### TBE Vaccine Safety

Although the TBE vaccine was only licensed in the United States in 2021, it is available in approximately 30 other countries, primarily in Europe, and during 2001–2021 at least 75 million doses of the current formulation of the vaccine were administered (*251*). On the basis of this experience, the TBE vaccine is considered to have a good safety profile, with adverse events reported more commonly after the primary series doses than after a booster dose (*265*).

#### Common Local and Systemic Adverse Events with TBE Vaccine in Adults

In clinical studies submitted for the vaccine’s licensure in the United States, 4,427 persons aged ≥16 years received at least one 0.5-mL dose of the TBE vaccine. The pivotal safety study was a single blind RCT conducted among adults aged 16–64 years in which persons were randomized to receive 2 doses of the TBE vaccine administered 21–35 days apart; in a follow-up study, persons received the third dose 6 months after dose 1 (*224*,*244*,*245*). Persons in the control group received another non–U.S.-licensed TBE vaccine. Study participants filled in diaries to document solicited local and systemic reactions for 4 days after each vaccine dose. A total of 2,977 persons received ≥1 dose of the U.S-licensed TBE vaccine. The most common reported solicited local reactions within 4 days of vaccination with the U.S.-licensed vaccine were injection site tenderness (30%) and pain (13%). The most common reported solicited systemic reactions were fatigue (6%), headache (6%), and myalgia (5%). In a later study after a booster dose administered 3 years after the primary series, the most common reported local reactions within 4 days of the booster dose among 240 adults were injection site tenderness (5%) and pain (4%) (*224*,*245*,*246*). The most common reported systemic reactions after the booster dose were headache, muscle pain, and malaise (all <1%).

#### Common Local and Systemic Adverse Events with TBE Vaccine in Children

In clinical studies submitted for vaccine licensure in the United States, 3,240 children aged 1–15 years received at least one 0.25-mL dose of the TBE vaccine. The pivotal pediatric safety study was an observational study that measured rates of adverse events after each of the 3 doses in the primary series (*224*,*247*). Caregivers of study participants filled in study diaries to document solicited local and systemic reactions for 4 days after each vaccine dose. Among 2,417 children or adolescents aged 1–15 years who received ≥1 vaccine dose, the most common local reactions reported within 4 days of a dose were injection site tenderness (18%) and pain (11%). The most common systemic reactions were headache (11%) and fever (10%). In addition, restlessness was reported among 9% (53 of 584) children aged 1–5 years after dose 1. Rates of fever were analyzed by age group. After the first dose of TBE vaccine, a fever occurred in 36% (66 of 186) of children aged 1–2 years, 13% (71 of 563) of those aged 3–6 years, and 6% (95 of 1,668) of those aged 7–15 years. Approximately half of all fevers were 100.4°F–101.1°F (38°C–38.4°C), and none were >104°F (40°C). Fever rates were three- to fivefold lower in each age group after doses 2 and 3. In a later booster dose study among 156 children, the most common local reactions were injection site pain (15%) and tenderness (10%) (*224*,*248*). The most common systemic reactions were headache (3%) and myalgia (3%). Fever was not reported among children or adolescents after the booster dose.

#### Serious Adverse Events After TBE vaccine

Serious adverse event (SAE) data were reported in 16 studies including four RCTs, nine observational studies, and three postmarketing surveillance studies (*219*). Among 3,562 adult and 3,350 pediatric participants who received ≥1 dose of vaccine in the primary series, no SAEs were considered vaccine related. Among 240 adult and 202 pediatric participants who received a booster dose of vaccine, no SAEs were considered vaccine related. Among 687 adult and 1,992 pediatric participants who received a primary series dose or booster dose in three active postmarketing surveillance studies, one SAE was considered possibly vaccine related by study investigators. A child aged 12 months with concomitant rhinopharyngitis, gastroenteritis, and otitis media diagnosed postvaccination was hospitalized for fever at 1 day after vaccination and had a febrile convulsion the following day (*266*). Although published case reports have occasionally suggested potential vaccine-related SAEs, the absence of reported concerns overall from Europe where millions of doses have been administered indicates SAEs after the TBE vaccine are likely rare (*267*,*268*).

### Vaccination of Pregnant or Breastfeeding Persons

No adequate, controlled human studies have investigated the safety or immunogenicity of TBE vaccine in pregnant persons, and no animal developmental and reproductive toxicity studies have been conducted. Multiple European countries have recommendations that indicate vaccine can be administered during pregnancy if clearly indicated based on risk (*269*). Transplacental transfer of TBE virus antibody from vaccinated mothers has been reported and is likely protective for an infant (*270*). ACIP best practice guidelines for vaccination indicate that no evidence exists for risk to the fetus from vaccinating pregnant women with inactivated viral vaccines (*271*).

No studies have assessed the safety or immunogenicity of TBE vaccine in breastfeeding persons, and no data are available on excretion in human milk. No inactivated vaccine has been indicated to cause harm in a breastfeeding infant (*272*). ACIP best practice guidelines for vaccination indicate inactivated vaccines administered to breastfeeding women do not affect the safety of breastfeeding for these women or their infants (*271*).

### Cost-Effectiveness of TBE Vaccine

Among populations in areas where TBE is endemic, results of cost-effectiveness analyses have been variable. TBE vaccination often has been considered not to be cost-effective, with incidence and vaccine cost being important variables in predicting cost-effectiveness (*196*,*273*–*275*). Cost-effectiveness analyses for TBE vaccination of travelers have not been conducted. On the basis of the low risk for disease and the vaccine’s cost, vaccination would not be expected to be cost-effective for U.S travelers. However, cost-effectiveness considerations are less relevant when vaccination is for an individual traveler rather than the population. Travel vaccines are not covered by the Vaccines for Children program and usually are not covered by insurance. Travelers’ decisions on purchasing vaccine will likely be based on their willingness and ability to pay and personal perceptions and tolerance of risk.

## TBE Prevention and Vaccine Recommendations

### Rationale for TBE Vaccine Recommendations for Persons Who Travel Abroad

For most U.S. travelers to areas where TBE is endemic, the risk for TBE is low. However, certain persons who travel abroad are at increased risk for infection because of the season and location of travel and their activities. The risk for exposure to infected ticks is highest for persons who will be in areas where TBE is endemic during the main transmission season (April–November) and who are planning to undertake recreational activities in woodland habitats (e.g., hiking, camping, cycling, hunting, fishing, birdwatching, or collecting mushrooms or berries) or who might be occupationally exposed (e.g., farmers, forestry workers, field researchers, and military personnel) (Box 2).

Taking measures to reduce the risk for tick bites decreases the risk for infection. TBE vaccine can further reduce infection risk and might be indicated for certain persons who are at higher risk for TBE. The risk-benefit assessment for vaccination should consider multiple factors, including the likelihood of exposure to TBE virus-infected ticks on the basis of activities and itinerary (e.g., location, rurality, season, and duration of travel or residence). Persons with extensive exposure to ticks are likely to be at highest risk. Extensive exposure can be considered based on the duration of travel and frequency of exposure and might include shorter-term (e.g., <1 month) travelers with daily or frequent exposure or longer-term travelers with regular (e.g., a few times a month) exposure to environments that might harbor infected ticks. Other risk-benefit considerations should include the 1) rare occurrence but potentially high morbidity and mortality of TBE, 2) higher risk for severe disease among certain persons (e.g., persons aged ≥60 years), 3) availability of an effective vaccine, 4) possibility but low probability of SAEs after vaccination, 5) likelihood of future travel to areas where TBE is endemic, and 6) personal perception and tolerance of risk.

### Recommendations for Prevention of TBE Among Persons Who Travel Abroad

#### Personal Protective Measures

All persons who travel to areas where TBE is endemic should be advised to take precautions to avoid tick bites. Preventive measures include using insect repellent registered by the Environmental Protection Agency, wearing protective clothing, and inspecting the body and clothing for ticks during and after outdoor activities. Travelers to areas where TBE is endemic also should be advised to avoid the consumption of unpasteurized dairy products.

#### Recommendations for the Use of TBE Vaccine Among Persons Who Travel Abroad

- TBE vaccine is recommended for persons who are moving or traveling to an area where TBE is endemic and will have extensive exposure to ticks because of their planned outdoor activities and itinerary.
- TBE vaccine may be considered for persons traveling or moving to an area where TBE is endemic who might engage in outdoor activities in areas where ticks are likely to be found. The decision to vaccinate should be based on an assessment of their planned activities and itinerary, risk factors for a poor medical outcome, and personal perception and tolerance of risk.

An algorithm to assist health care providers with decision-making for TBE vaccination for U.S. travelers is provided (Figure). The evidence for these recommendations can be found in the GRADE and EtR documents available at https://www.cdc.gov/vaccines/acip/recs/grade/table-refs.html and https://www.cdc.gov/vaccines/acip/recs/grade/etr.html.

**FIGURE:**
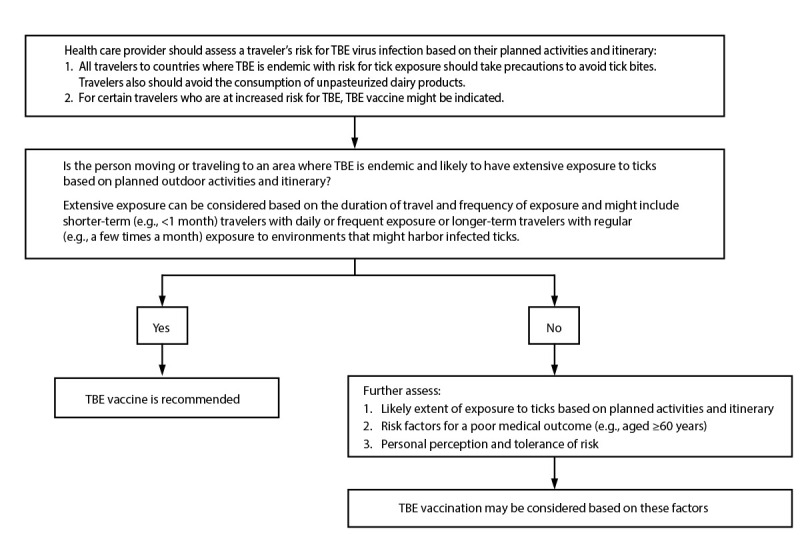
Decision-making for recommending tick-borne encephalitis vaccination for U.S. travelers to areas where the disease is endemic **Abbreviation:** TBE = tick-borne encephalitis.

### Recommendations for Prevention of TBE Among Laboratory Workers

TBE virus transmission through aerosolization has been documented in the laboratory setting, and transmission through accidental percutaneous or mucosal exposures is possible. Although no published reports of laboratory-associated TBE virus infections in vaccinated laboratory workers are available, the level of protection that TBE vaccination provides against infection via these transmission routes is unknown.

#### Best Practices for Working with TBE Virus in the Laboratory

Laboratory work with TBE virus is generally restricted to BSL-4 facilities and practices. Recommendations for best practices for the safe conduct of work in biomedical and clinical laboratories are available (*217*).

#### Recommendations for the Use of TBE Vaccine Among Laboratory Workers

- TBE vaccine is recommended for laboratory workers with a potential for exposure to TBE virus.

A local institutional biosafety committee should undertake a risk assessment of the potential for exposure to TBE virus for each laboratory worker working with TBE virus. The committee should consider the type of work to be performed and the biosafety level at which work will be conducted. Vaccination is not required for workers handling routine clinical samples.

Laboratory workers should receive a standard 3-dose primary series and 1 booster dose. If the potential for ongoing laboratory exposure after receipt of these doses exists, a local biosafety committee should provide guidance on the need for periodic checks of TBE virus-specific neutralizing antibody titers.

## Administration of TBE Vaccine

### Vaccine Composition, Presentation, Storage, and Handling

The TBE vaccine is an inactivated, whole virus vaccine prepared using a European subtype of TBE virus called the Neudoerfl strain, which was originally isolated from ticks in Austria (*230*). The vaccine is available as a 0.5-mL presentation for persons aged ≥16 years and a 0.25-mL presentation for children and adolescents aged 1–15 years (*224*). Each 0.5-mL dose contains 2.4 *μ*g, and each 0.25-mL dose contains 1.2 *μ*g of inactivated TBE virus. The vaccine is produced on chick embryo fibroblast cells with aluminum hydroxide as an adjuvant and human serum albumin as a stabilizer. No thimerosal or other preservatives are included. A list of additional components and substances used in manufacturing that might be found in the final product is available (Table 3).

**TABLE 3:** Characteristics and properties of tick-borne encephalitis vaccine

Vaccine characteristic	Vaccine property
Trade name	Ticovac
Vaccine type	Inactivated, whole virus
TBE virus strain	Neudoerfl (European TBE virus subtype)
Substrate	Chick embryo fibroblast cells
Adjuvant	Aluminum hydroxide
Stabilizer	Human serum albumin
Preservative	None
Other constituents	Sodium chloride, dibasic sodium phosphate, and monobasic potassium phosphate
Substances used in manufacturing that might be present in final product	Formaldehyde; sucrose; protamine sulfate; trace amounts of neomycin, gentamicin, and chick protein; and DNA from chick embryo fibroblast cells
Final preparation	Suspension for injection
Presentation	Prefilled syringe
Storage	36°F–46°F (2°C–8°C)
Route	Intramuscular
Dose by age group	Aged ≥16 years: 0.5 mL (adult presentation) Aged 1–15 years: 0.25 mL (pediatric presentation)
Primary schedule by age group	Aged ≥16 years: 3 doses administered as follows: Dose 1: On day 0 Dose 2: 14 days–3 months after dose 1 Dose 3: 5–12 months after dose 2 Aged 1–15 years: 3 doses administered as follows: Dose 1: On day 0 Dose 2: 1–3 months after dose 1 Dose 3: 5–12 months after dose 2
Booster dose	All age groups: ≥3 years after completion of the primary series (if ongoing exposure or re-exposure to TBE virus is expected). No Advisory Committee on Immunization Practices recommendations are made on the need for subsequent booster doses.

**Abbreviation**: TBE = tick-borne encephalitis.

TBE vaccine is supplied in a prefilled syringe. The tip cap and plunger are not made with natural rubber latex. The vaccine should be stored at 36°F–46°F (2°C–8°C) and should not be frozen. It should be protected from light. Before administration, the vaccine should be brought to room temperature and shaken well. After shaking, it should be an off-white, homogenous, opalescent suspension. Vaccine should not be administered if particulate matter or discoloration remains after shaking.

### Dosage, Schedule, and Administration

#### Primary Vaccination Series

The vaccination dose and timing for the 3-dose primary TBE vaccine series vary by age (Table 3).

- **1–15 years:** 3 doses (0.25 mL each) administered intramuscularly (IM) with the first 2 doses administered 1–3 months apart and the third dose administered 5–12 months after dose 2.
- **≥16 years:** 3 doses (0.5 mL each) administered IM with the first 2 doses administered 14 days to 3 months apart and the third dose administered 5–12 months after dose 2.

For all age groups, the 3-dose primary vaccination series should be completed at least 1 week before potential exposure to TBE virus. For persons (e.g., travelers) who cannot complete the 3-dose primary series, see Immunologic Response After an Incomplete Primary Series (1 or 2 Doses) in Adults and Children.

#### Booster Dose

For all age groups, a booster dose (i.e., fourth dose) can be administered at least 3 years after completion of the primary 3-dose TBE vaccine series if ongoing exposure or re-exposure to TBE virus is expected. No ACIP recommendations are made on the need for subsequent booster doses.

- **1–15 years:** 0.25-mL dose administered IM ≥3 years after completion of primary series.
- **≥16 years:** 0.5-mL dose administered IM ≥3 years after completion of primary series.

#### Concomitant Administration of Other Vaccines or Drugs

Data are limited on co-administration of TBE vaccine with other vaccines or drugs. One clinical trial in which the first dose of TBE vaccine was administered concomitantly with yellow fever vaccine indicated no interference with the immune response to TBE vaccine or yellow fever vaccine (*264*). If TBE vaccine and other vaccines are administered concomitantly, they should be administered with separate syringes and at different anatomic sites (*271*).

## Contraindications for the Use of TBE Vaccine

### Allergy to Vaccine Components

A severe allergic reaction (e.g., anaphylaxis) to any component of the TBE vaccine, including substances remaining from the manufacturing process (e.g., protamine sulfate, neomycin, gentamicin, and chick protein), is a contraindication to vaccination (*224*) (Table 3). Although TBE vaccine is prepared from TBE virus propagated in chick embryo fibroblast cells, the ultracentrifugation process removes all but trace amounts of chick protein. No reports have been published related to TBE vaccine and egg allergy. However, a known severe hypersensitivity to egg or chicken protein (e.g., anaphylaxis after oral ingestion) is a contraindication to TBE vaccination. As with any vaccine, all persons should be vaccinated in settings with the capacity to manage allergic reactions (*271*).

## Precautions for the Use of TBE Vaccine

### Altered Immune States

Having an immunocompromising condition or being immunosuppressed are precautions for vaccination. The immunogenicity and safety of TBE vaccine in persons with altered immune status has not been well characterized. Available information suggests the vaccine can be safely administered, but the immune response might be diminished (*224*,*276*–*278*). Additional information on vaccination in persons with altered immunocompetence can be found in the ACIP general guidelines for best vaccination practices (*271*).

### Special Populations

**Pregnant persons:** Pregnancy is not a contraindication or precaution to vaccination. TBE virus infection can pose a risk for severe illness in pregnant persons; thus, the benefits of vaccinating pregnant persons when the likelihood of infection is high likely outweigh the potential risks.

**Breastfeeding persons:** Breastfeeding is not a contraindication or precaution to vaccination with TBE vaccine.

**Infants aged <1 year:** Safety and effectiveness of TBE vaccine have not been established for infants aged <1 year.

**Persons in older age groups:** Data on safety and immunogenicity of TBE vaccine among persons aged >50 years are limited but suggest there are no safety concerns. Immunologic data suggest the response to TBE vaccine is reduced among adults aged >50 years, particularly among those aged ≥65 years. Levels of neutralizing antibodies are lower postvaccination in older adults, and older adults are more likely to become seronegative than younger adults in the years after their last dose of vaccine.

## Reporting of Vaccine Adverse Events

Surveillance for adverse events associated with administration of TBE vaccine is important. An adverse event that occurs after vaccine administration, even if a causal relation to vaccination is not certain, should be reported to the Vaccine Adverse Event Reporting System (VAERS) at https://vaers.hhs.gov or by calling 1-800-822-7967.

## Future Research

The ACIP Tick-Borne Encephalitis Vaccine Work Group identified multiple key areas for further research. One important topic is the level of protection afforded by the U.S.-licensed TBE vaccine, based on a European subtype TBE virus, for non-European TBE virus subtypes.

Evaluation of potential immunological interactions between TBE vaccine and other flaviviruses or flavivirus vaccines (e.g., yellow fever or Japanese encephalitis vaccines) is needed. A small study investigating the effect of previous yellow fever vaccination found that TBE virus GMTs were significantly lower in persons who previously received yellow fever vaccine compared with flavivirus-naïve persons (*279*). In vaccine trials, persons with evidence of previous flavivirus exposure were usually excluded; therefore, determining whether previous exposure to other flaviviruses endemic in the United States (e.g., the tickborne Powassan virus or the mosquitoborne West Nile virus) or to flaviviruses travelers might have been exposed to (e.g., dengue and Zika viruses) might affect the immune response to TBE vaccine would be useful. Likelihood of interference is low when TBE vaccine is administered simultaneously with other vaccines, medications, or substances because it is an inactivated vaccine; however, potential immunological interactions could be addressed by appropriate studies.

Data are limited on TBE vaccine use among certain populations. Data on immunogenicity of TBE vaccine among older adults are limited and suggest the immunologic response to TBE vaccine is reduced. Additional data on vaccine protection among older adults would be valuable, particularly the need for and timing of any additional booster doses. Further research is warranted to assess the clinical spectrum of TBE virus infection during pregnancy to better understand the extent of risk for this population. Although no data are available to suggest concerns with use of this inactivated vaccine among pregnant or breastfeeding persons, systematic collection of additional data in these populations would be beneficial.

## Additional Information

Additional information about TBE is available from CDC at https://www.cdc.gov/tick-borne-encephalitis. Additional licensure information for TBE vaccine is available from FDA (https://www.fda.gov/vaccines-blood-biologics/ticovac).
